# Can Phosphite Substitute for Phosphate in ptxD Rice? Evidence From Uptake and Agronomic Performance

**DOI:** 10.1111/ppl.70969

**Published:** 2026-06-19

**Authors:** Clenya Carla Leandro de Oliveira, André Luís da Silva Parente Nogueira, José Nivaldo de Oliveira Sátiro, Tiago Paula da Silva, Maria Eduarda Pimentel de Melo, Castro Alves da Silva Junior, Izabela Gouveia Nascimento, Natália Corrêa da Silva, Gabriel Lima Leal, Marcelo de Freitas Lima, Andrés Calderín García, Leandro Azevedo Santos

**Affiliations:** ^1^ Institute of Agronomy, Department of Soil Science Federal Rural University of Rio de Janeiro Seropédica Brazil; ^2^ Institute of Biomedical Sciences, Federal University of Rio de Janeiro Rio de Janeiro Brazil; ^3^ Institute of Chemistry, Department of Biochemistry Federal Rural University of Rio de Janeiro Seropédica Brazil

**Keywords:** alternative phosphorus source, phosphate fertilization, phosphite uptake

## Abstract

Engineering crops to metabolize phosphite (Phi) as a phosphorus (P) source has been reported as a promising strategy to enhance nutrient use efficiency and enable innovative crop management. Here, we evaluated rice ( *Oryza sativa* L.) plants engineered with a codon‐optimized ptxD gene from *Pseudomonas stutzeri* for their capacity to utilize Phi under soil and hydroponic conditions. Two independent transgenic lines (L1 and L2), constitutively expressing ptxD, were cultivated in a Ferralsol with low available P and supplied with phosphate (Pi) or phosphite (Phi) at graded fertilization doses. Parallel hydroponic trials tested a wide range of P concentrations. To monitor uptake dynamics with high precision, we applied a fluorometric assay based on thermostable phosphite dehydrogenase (17X‐PTDH), enabling real‐time quantification of Pi and Phi depletion. Under Phi fertilization, transgenic lines displayed enhanced growth and phosphorus accumulation compared with wild‐type (WT) plants, confirming the functional expression of ptxD. However, agronomic performance with Phi was consistently lower than with Pi, especially at reduced doses. Uptake kinetics revealed slower Phi absorption even following P starvation, and biomass accumulation was restricted at low Phi concentrations. This work provides the first direct evidence, obtained through depletion assays, that Phi acquisition is intrinsically less efficient than Pi uptake. Together, these findings establish that while ptxD rice can metabolize Phi as a P source, its acquisition efficiency remains substantially inferior to Pi. Phi should therefore be regarded as a complementary fertilizer or as a selective tool for weed management in ptxD‐based systems, rather than a complete substitute for phosphate.

## 1 Introduction

Rice ( *Oryza sativa* L.) is a cornerstone of global food security, serving as the primary dietary staple for billions of people, particularly in Asia and Latin America (da Silva and Wander 2025). In Brazil, it represents a critical food source, especially for vulnerable populations (FAO 2022; USDA 2023). However, rice productivity is often constrained by environmental limitations, particularly in rainfed systems established on highly weathered oxidic soils, which are typically acidic and characterized by low phosphorus (P) availability (Vinha et al. 2021). Phosphorus is essential for plant development, as it is required for energy transfer and the biosynthesis of key biomolecules such as nucleic acids and phospholipids (Han et al. 2021).

Globally, nearly 90% of mined phosphorus is used in fertilizer production (Pantano et al. 2016). Yet, fertilizer‐use efficiency in tropical soils such as Ferralsols remains notoriously low due to strong P fixation through adsorption and precipitation with iron (Fe) and aluminum (Al) oxides, which are abundant in these environments (Juo and Franzluebbers 2003; Lambers 2022). Consequently, plants typically absorb only 10%–20% of the applied P (Roberts and Johnston 2015), while the remainder is immobilized in the mineral fraction or incorporated into the organic pool via microbial activity (Luz and Brito 2022).

Phosphite (Phi), a reduced form of phosphorus, was first considered in the 1950s as a potential alternative fertilizer due to its higher solubility and lower reactivity with soil components (Manna et al. 2016). Although Phi is readily taken up by plants, it cannot be metabolized by them (Navea et al. 2024), restricting its use as a conventional P source. However, certain bacteria, such as *Pseudomonas stutzeri* WM88, harbor the *ptxD* gene, which encodes phosphite dehydrogenase (ptxD), an enzyme that oxidizes Phi into phosphate (Pi) (Costas et al. 2001; White and Metcalf 2007). Heterologous expression of *ptxD* in plants enables the metabolic conversion of Phi to Pi, allowing plants to use Phi as a phosphorus source (López‐Arredondo and Herrera‐Estrella 2012).

Several studies have shown that *ptxD*‐expressing plants supplied with Phi can achieve performance levels comparable to those obtained with Pi, suggesting a functional equivalence between the two sources (Manna et al. 2016; Xu et al. 2024). Nonetheless, critical gaps remain regarding the factors that determine Phi‐use efficiency, particularly the uptake process and the subsequent enzymatic conversion to Pi.

Recent advances have focused on engineering more efficient ptxD variants through codon optimization, site‐directed mutagenesis, and directed evolution. For instance, a variant termed ptxD_Q displayed a 5.8‐fold increase in catalytic efficiency, enhancing Phi utilization in Arabidopsis and rice (Liu et al. 2021). Similarly, mutations in *Chlamydomonas reinhardtii* ptxD broadened cofactor specificity, enabling the use of both NAD^+^ and NADP^+^ and promoting faster phototrophic growth (Cutolo et al. 2020). These strategies aim to overcome potential bottlenecks in Phi‐to‐Pi conversion, thereby improving overall system performance.

Despite these advances, limitations in Phi utilization may extend beyond enzymatic conversion, involving constraints at the level of Phi uptake by plant cells. Here, we generated genetically modified rice plants constitutively expressing a codon‐optimized *ptxD* gene from *Pseudomonas stutzeri* WM88, and evaluated their capacity to absorb and metabolize Phi as a phosphorus source under oxidic soil conditions and in nutrient solution. This study provides new insights into the physiological and biochemical constraints of Phi‐based fertilization and its potential as a sustainable agricultural strategy.

## 2 Materials and Methods

### 2.1 Genetic Construct Preparation and Rice ( *Oryza sativa* L.) Transformation

The *ptxD* gene from *Pseudomonas stutzeri* WM88 (GenBank: AF061070.1) was synthesized with codons optimized for rice ( *Oryza sativa* L. ssp. *japonica*) (Figure S1). The expression vector IRS154 (kindly provided by Emmanuel Guiderdoni and Martine Bes, CIRAD, France), which is identical to PC1300intA.Ubi‐tNos, was used as the backbone. The *ptxD* gene was amplified using hybrid primers containing BamHI and KpnI restriction sites. Both the amplicon and IRS154 vector were digested with the corresponding enzymes and subsequently ligated using T4 DNA ligase, according to the manufacturer's instructions (Promega).

Construct integrity was confirmed by Sanger sequencing, and the recombinant vector (Figure S2) was introduced into *Agrobacterium tumefaciens* strain LBA4404 via heat‐shock transformation (Holsters et al. 1978). Rice ( *O. sativa* cv. Nipponbare) transformation was performed following the protocol described by Toki et al. (2006), and the transformed plants were subsequently confirmed by PCR analysis (Figure S3; Table S1). Transgenic lines were selected based on their ability to grow in a nutrient solution containing Phi as the sole phosphorus source. Two lines that exhibited stable growth under Phi and a Mendelian 3:1 segregation ratio for the transgene were advanced. Homozygous T2 seeds from these lines were used for subsequent experiments.

### 2.2 Ferralsol Experiment

To assess the efficiency of phosphate fertilization using inorganic phosphate (Pi) or Phi in ptxD‐expressing rice, a soil experiment was conducted until plant maturity. The aim was to compare agronomic performance and evaluate the feasibility of Phi as an alternative to Pi in ptxD rice.

A Ferralsol (WRB 2022) was collected from the 0–20 cm soil layer in Pinheiral, Rio de Janeiro, Brazil (22°35′55″ S, 43°56′27″ W). The soil was air‐dried, sieved through a 4 mm mesh, and subjected to chemical (Table 1) and physical characterization according to Teixeira et al. (2017). Particle size distribution revealed the following composition: 481 g kg^−1^ clay, 114 g kg^−1^ silt, and 405 g kg^−1^ sand.

**TABLE 1:** Chemical attributes of Ferralsol samples collected at 0–20 cm depth.

pH (H_2_O)	C.O. g kg^−1^	P	SO_4_^−2^	Na	K	Ca	Mg	H + Al	*S*	*T*	*V*	*m*
		mg dm^−3^		cmolc dm^−3^							%	
4.40	38.0	3.7	16.00	0.22	0.11	1.7	0.6	5.9	2.64	8.54	31.0	18.5

Abbreviations: C.O.: organic carbon; *m*: aluminum saturation percentage; *S*: sum of exchangeable bases; *T*: total cation exchange capacity; *V*: base saturation percentage.

The soil used in the experiment was considered suitable, as chemical analysis revealed a very low level of available phosphorus (3.7 mg dm^−3^). In addition, it exhibited high acidity (pH 4.4) and a high concentration of exchangeable aluminum (Al^3+^ = 1.3 cmolc dm^−3^), which required liming. Soil acidity was corrected with dolomitic limestone (PRNT 83.1%) applied at a rate of 0.884 g L^−1^ of soil, equivalent to 1.77 Mg ha^−1^.

Seeds of wild‐type (WT), L1, and L2 rice plants were germinated on gauze in containers filled with distilled water. Seven days after germination (DAG), two seedlings were transplanted into each pot containing 8 kg of pre‐limed soil. Plants were grown under four phosphorus fertilization levels (0%, 25%, 50%, and 100% of the recommended rate), supplied either as inorganic phosphate (Pi) or Phi. The experiment followed a randomized complete block design with four replicates per treatment, totaling 96 experimental units (Figure S4). Based on soil chemical analysis, the recommended fertilization rates were 35 kg ha^−1^ of P (full P dose), 90 kg ha^−1^ of N, and 60 kg ha^−1^ of K, in accordance with the Soil Fertility Manual (Freire 2013). For the pot experiment, these rates were adjusted according to soil mass, assuming a fertilization depth of 0.20 m, which corresponds to 2,000,000 dm^3^ of soil per hectare. Considering a soil bulk density of 1.15 kg dm^−3^ and 8 kg of soil per pot (≈7 dm^3^), the equivalent rates were 0.1225 g P (full P dose), 0.315 g N, and 0.210 g K per pot. Nitrogen was supplied as urea (46.7% N), potassium as KCl (52.4% K), and phosphorus as phosphate (Pi, Na_2_HPO_4_, 21.8% P) or Phi (Na_2_HPO_3_·5H_2_O, 14.3% P), depending on the treatment (Table S3). In plants grown under different Pi and Phi fertilization levels, the following agronomic traits were assessed: biomass accumulation, tiller number, panicle number, and grain yield.

### 2.3 Hydroponic Assay With Different Pi and Phi Levels

This experiment was designed to evaluate the performance of the ptxD L1 line under varying concentrations of Pi and Phi to infer the relative uptake efficiency of these phosphorus sources. In preliminary soil and hydroponic‐based assays, both transgenic lines (L1 and L2) showed similar growth patterns and comparable responses to Pi and Phi supply, with no consistent differences in P‐related traits. Therefore, L1 was selected as a representative line for the present detailed dose–response experiment. Seed germination and plant cultivation were carried out in a greenhouse during spring in Seropédica, Rio de Janeiro, Brazil (22°45′33.7″ S, 43°41′51.1″ W), with a maximum temperature of 28°C regulated by an evaporative cooling system and a 12‐h photoperiod. Seeds of WT and L1 plants were surface‐sterilized in 2.0% sodium hypochlorite solution, shaken for 20 min, and rinsed seven times with distilled water. Five DAG, seedlings were transferred to ¼‐strength Hoagland nutrient solution without phosphorus and maintained there until 10 DAG. Subsequently, plants were cultivated in ½‐strength Hoagland solution supplemented with either Phi or phosphate (Pi) at concentrations of 5, 10, 25, 200, and 500 μM. The nutrient solution was renewed every 3 days. Plants were harvested 28 DAG, a developmental stage corresponding to early vegetative growth with initial tillering, when the root system is fully established and phosphate uptake mechanisms are functionally active. This time point was selected to ensure morphological uniformity among plants and sufficient shoot and root biomass for reliable phenotypic measurements and dry mass determination, while avoiding potential interference from later developmental stages. Images were recorded, and shoot and root length were measured. Plant material was then placed in paper bags, oven‐dried at 60°C for 7 days, and weighed to determine shoot and root dry mass.

### 2.4 Hydroponic Assay for Gene Expression Analysis

This assay was conducted to evaluate the expression of genes associated with phosphorus acquisition and responses to phosphorus deficiency in WT plants and ptxD lines grown with either Pi or Phi. Germination and plant cultivation were performed in a growth chamber at the Department of Soil Science, UFRRJ, under a photosynthetic photon flux density of 318–330 μmol m^−2^ s^−1^, with a 14 h light/10 h dark photoperiod, 70% relative humidity, and day/night temperatures of 28°C/24°C. Seeds of WT, L1, and L2 plants were surface‐sterilized as described above.

Following sterilization, seeds were placed on gauze in 300 mL cups and germinated in ¼‐strength Hoagland nutrient solution supplemented with either 0.2 mM phosphate or 0.2 mM Phi. The phosphorus deprivation treatment was applied only to WT plants to verify that the experimental conditions were sufficient to trigger the canonical transcriptional response associated with Pi starvation. Nutrient solutions were renewed every 3 days. At 14 DAG, plants were harvested, and root samples were flash‐frozen in liquid nitrogen and stored at −80°C for subsequent analyses. Total RNA was extracted following the protocol of Gao et al. (2001) and quantified using a NanoDrop spectrophotometer (Thermo Scientific). RNA quality was assessed by A_260/230_ and A_260/280_ ratios (ranging from 1.9 to 2.1), and integrity was confirmed by electrophoresis on 1% agarose gel stained with GelRed. One microgram of total RNA was treated with DNase I Amplification Grade (Sigma‐Aldrich) to remove genomic DNA, and cDNA was synthesized using the High‐Capacity RNA‐to‐cDNA kit (Thermo Scientific) according to the manufacturer's instructions.

Quantitative real‐time PCR was performed in a StepOne Plus Real‐Time PCR System (Applied Biosystems) using Power SYBR Green PCR Master Mix (Applied Biosystems). The thermal cycling program consisted of an initial denaturation at 95°C for 15 min, followed by 40 cycles of 95°C for 15 s and 60°C for 1 min. Relative gene expression was calculated using the comparative Ct method (2^−∆∆Ct^) (Livak and Schmittgen 2001), with *OsACT1* (rice actin 1) serving as the endogenous reference gene. Primer sequences used in this study are provided in Table S2.

### 2.5 Pi and Phi Depletion Kinetics

This assay was designed to evaluate the dynamics of Pi and Phi uptake in WT plants and ptxD lines L1 and L2 to identify potential differences in absorption rates that could provide insights into the affinity of Pi transporters for these two phosphorus sources. Seed sterilization, germination, and growth were conducted as described previously. At five DAG, seedlings were transferred to 1 L pots containing ¼‐strength Hoagland nutrient solution supplemented with 0.1 mM Pi and maintained until 8 DAG. Plants were then supplied with ½‐strength Hoagland solution containing 0.1 mM Pi for 2 weeks, with solution renewal every 3 days. Following this period, plants were transferred to phosphorus‐free solution for 6 days, corresponding to two consecutive renewals of P‐free solution. Subsequently, plants were divided into three treatment groups and supplied with nutrient solutions containing: (i) 0.2 mM Pi only, (ii) 0.2 mM Phi only, or (iii) a mixture of Pi and Phi at 0.1 mM each. To monitor nutrient depletion, 1 mL aliquots of nutrient solution were collected every 2 h during the first 12 h, with additional samples taken at 26 and 28 h.

Nutrient uptake rates were calculated from the linear phase of the depletion curves (0–10 h). For each replicate, nutrient concentration over time was fitted by linear regression, and the slope (b; dC/dt, μmol L^−1^ h^−1^) was used to estimate uptake rate (J) according to the equation:

$$J=-b\times \frac{V}{M_{r}}$$

where *J* is the Pi or Phi uptake rate (μmol g^−1^ h^−1^), *V* is the volume of the nutrient solution (L), and *M*_r_ is the root dry mass (g). The negative sign accounts for the decrease in nutrient concentration over time.

Phosphorus accumulation in shoots and roots of WT and ptxD (L1) plants was determined after harvesting and drying the tissues from each pot containing four plants. Ground samples were subjected to acid digestion, and total P concentration was quantified by a colorimetric assay based on phosphomolybdate complex formation, as previously described (Tedesco et al. 1995). Total P accumulation per pot was calculated by multiplying tissue P concentration by the corresponding dry mass.

### 2.6 PTDH‐17x Expression and Purification

Phi quantification was performed enzymatically as described by Bailey and Greene (2023), using 17X‐PTDH, a thermostable variant of PTDH (Howe and Van Der Donk 2019). The plasmid pET15b‐17X‐PTDH was kindly provided by Wilfred van der Donk (Addgene plasmid #166786; http://n2t.net/addgene:166786; RRID: Addgene_166786) (Yang and van der Donk 2015). *Escherichia coli* BL21 (DE3) cells were transformed with pET15b‐17X‐PTDH, and a single colony was inoculated into 1 L of LB medium supplemented with 100 μg mL^−1^ ampicillin and 100 mM glucose. Cultures were grown at 37°C with shaking at 200 rpm until the optical density at 600 nm (OD_600_) reached 0.6. Protein expression was induced with 0.5 mM IPTG, and cultures were incubated at 18°C under constant agitation for 18 h. Cells were harvested by centrifugation (4000 ×*g*, 4°C, 10 min), and the pellet was resuspended in Buffer A (20 mM Tris‐Cl [pH 7.6], 0.5 M NaCl, 10% [v/v] glycerol). The suspension was incubated with 0.5 mg mL^−1^ lysozyme (Sigma‐Aldrich) and 6.6 U mL^−1^ DNase (Thermo Fisher) at 4°C for 1 h, followed by sonication for 40 min using 10 W pulses (15 s on/15 s off, 40% amplitude; Qsonica Sonicators). The lysate was clarified by centrifugation (16,000 ×*g*, 4°C, 1 h) and applied to a HisPur Ni‐NTA Spin column (Thermo Fisher) pre‐equilibrated with Buffer A. The column was washed with Buffer B (20 mM Tris‐Cl [pH 7.6], 0.5 M NaCl, 10% [v/v] glycerol, 25 mM imidazole) and eluted with Buffer C (20 mM Tris‐Cl [pH 7.6], 0.5 M NaCl, 10% [v/v] glycerol, 500 mM imidazole). Fractions containing the target protein were concentrated using Amicon Ultra centrifugal filters (Merck, 10 kDa MWCO) and subjected to buffer exchange/desalting with a Zeba Spin Desalting column (Thermo Fisher) using Buffer D (20 mM MOPS [pH 7.6], 100 mM KCl, 10% [v/v] glycerol). Protein concentration was determined by the Bradford assay.

### 2.7 Phosphite and Phosphate Analysis

As reported by Bailey and Greene (2023), the composition of the reaction medium can influence PTDH activity. To account for this, the standard curve used to calculate Phi concentrations, ranging from 0 to 200 μM, was prepared in the same nutrient solution used for plant cultivation. Phi quantification assays were performed in 96‐well microplates, with a final reaction volume of 250 μL per well. Each reaction contained 200 μL of a mix consisting of 50 mM MOPS (pH 7.3), 100 μM NAD^+^, 100 μM phenazine methosulfate (PMS), 100 μM resazurin, and 1 μg of purified 17X‐PTDH, along with 50 μL of sample. Reactions were conducted in triplicate. Plates were incubated at 37°C, and the fluorescent product, resorufin, was quantified using a microplate reader (Varioskan LUX 3020‐8277; Thermo Fisher Scientific) at excitation and emission wavelengths of 535 and 585 nm, respectively. For phosphate (Pi) quantification, a standard curve ranging from 0 to 200 μM was prepared using KH_2_PO_4_. Phosphate content was determined using the ammonium molybdate colorimetric method, with absorbance measured at 660 nm, following the protocol described by Teixeira et al. (2017).

### 2.8 Statistical Analysis

All experiments were conducted in a randomized complete block design. Statistical analyses were performed using the R software environment (R Core Team 2020). Data were tested for normality and homogeneity of variances. Variables that did not meet the normality assumption were transformed using the Box–Cox method. When the assumptions were satisfied, analyses of variance (ANOVA) were performed, and means were compared using Tukey's test (*p* ≤ 0.05).

Factorial ANOVA models were applied according to each experimental design to evaluate main effects and interactions. The experiment conducted in Ferralsol soil (WRB 2022) followed a two‐factor factorial design (P source × [concentration + genotype]). The nutrient solution experiment was analyzed using a three‐factor factorial scheme (P source × concentration × genotype). Data were tested for normality and homogeneity of variances using the Shapiro–Wilk and Bartlett tests (both at *p* ≤ 0.05). Interaction effects were explicitly tested and, when significant, simple effects were compared using Tukey's multiple comparison test (*p* ≤ 0.05).

## 3 Results

**FIGURE 1:**
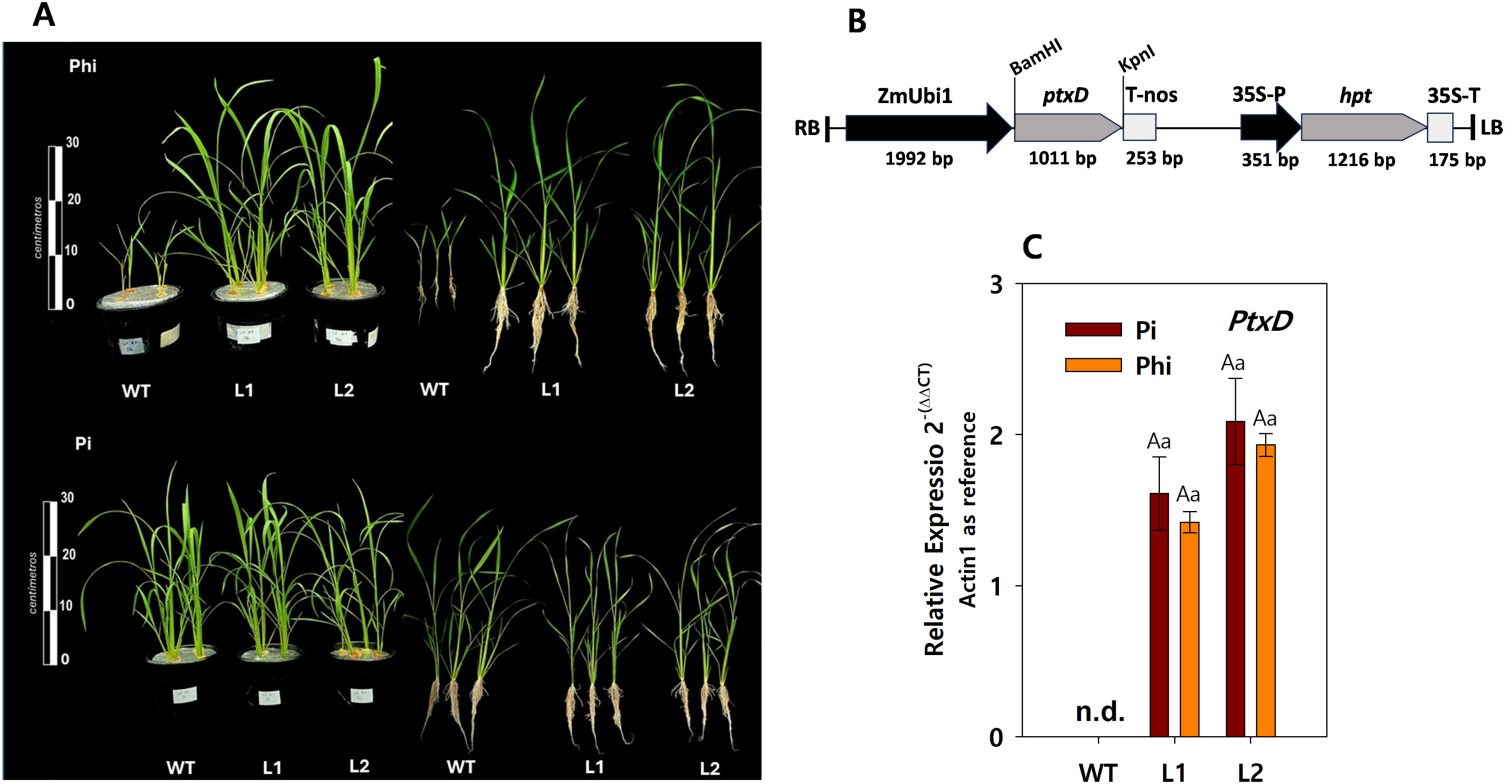
Development of WT plants and ptxD transgenic lines grown with phosphite (Phi) or phosphate (Pi), both at 0.2 mM. (A) Phenotype of WT and transgenic lines L1 and L2 at 14 DAG in hydroponic culture, with solution renewed every 3 days. (B) Schematic representation of the genetic construct used for plant transformation, containing the rice‐optimized *ptxD* gene under the control of the ZmUbi1 promoter. (C) Expression levels of *ptxD* in lines L1 and L2 grown with Phi or Pi, determined by qPCR and normalized to the reference gene *ACTIN 1*. Means followed by the same letters do not differ significantly. Capital letters compare P sources within each genotype, whereas lowercase letters compare genotypes within each P source, according to Tukey's test at the 5% significance level.

Two transgenic rice lines expressing the *ptxD* gene, designated L1 and L2, were selected based on their capacity to metabolize Phi and sustain growth in nutrient solution where Phi was the sole phosphorus source. WT plants and T2 homozygous plants of lines L1 and L2 were cultivated in ½‐strength Hoagland solution containing only Phi until 14 DAG (Figure 1).

**FIGURE 2:**
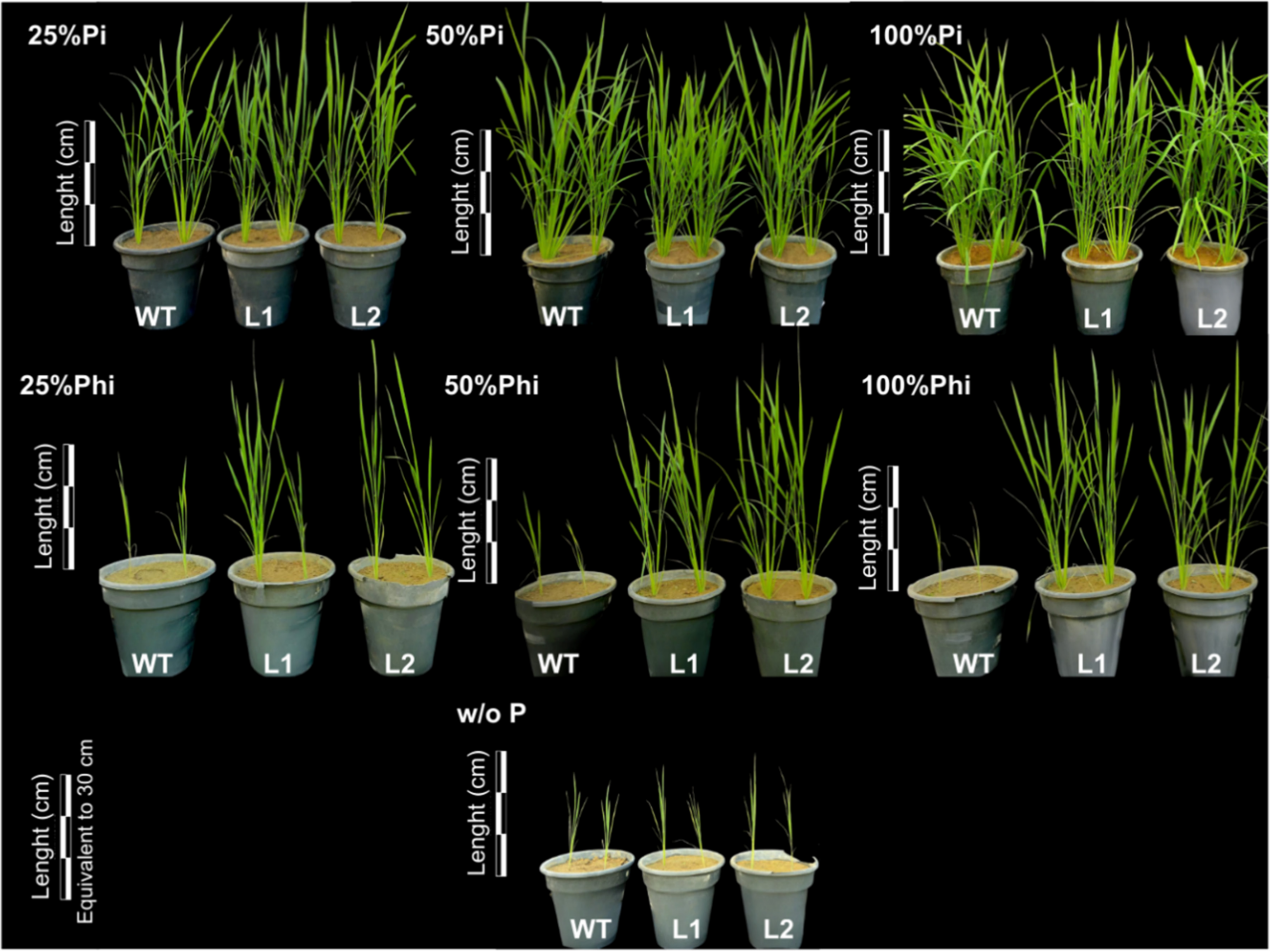
Development of WT plants and ptxD transgenic lines (L1 and L2) grown in Ferralsol fertilized with different doses of phosphite (Phi) or phosphate (Pi). Fertilizer doses corresponded to 0%, 25%, 50%, and 100% of the recommended phosphorus rate, equivalent to 0, 8.75, 17.5, and 35 kg ha^−1^ of elemental P. These rates corresponded to 4.36, 8.72, and 17.44 mg kg^−1^ of soil P. Pi was supplied as Na_2_HPO_4_ at 20, 40, and 80 mg kg^−1^ of soil, whereas Phi was supplied as Na_2_HPO_3_·5H_2_O at 30.41, 60.82, and 121.64 mg kg^−1^ of soil. Detailed stoichiometric calculations and the exact salt amounts applied per pot are provided in Table S3. Plants were evaluated 45 days after transplanting.

The *ptxD* gene, derived from *Pseudomonas stutzeri* WM88, was codon‐optimized for rice expression (Figure S1) and placed under the control of the constitutive ZmUbi1 promoter in the IRS154 expression vector (Figure S1). This configuration ensured high levels of transgene expression in the transgenic lines (Figure 2C), surpassing the expression of the endogenous actin 1 gene, which was used as the reference for normalization in quantitative expression analyses.

### 3.1 Performance of PtxD Lines Grown in Ferralsol Fertilized With Different Pi and Phi Levels

Using a Ferralsol with extremely low available phosphorus (3.7 mg P dm^−3^; Table 1), we observed that, in the absence of phosphorus fertilization, all plants exhibited severely stunted growth (Figure 2). At 45 days after transplanting, the growth of ptxD lines was substantially lower in soil supplemented with Phi compared to phosphate (Pi) (Figure 2). These results indicate that the capacity of ptxD lines to uptake and/or utilize Phi as the sole phosphorus source may be limited in soils with low residual Pi, or potentially influenced by other edaphic factors that remain to be elucidated.

**FIGURE 3:**
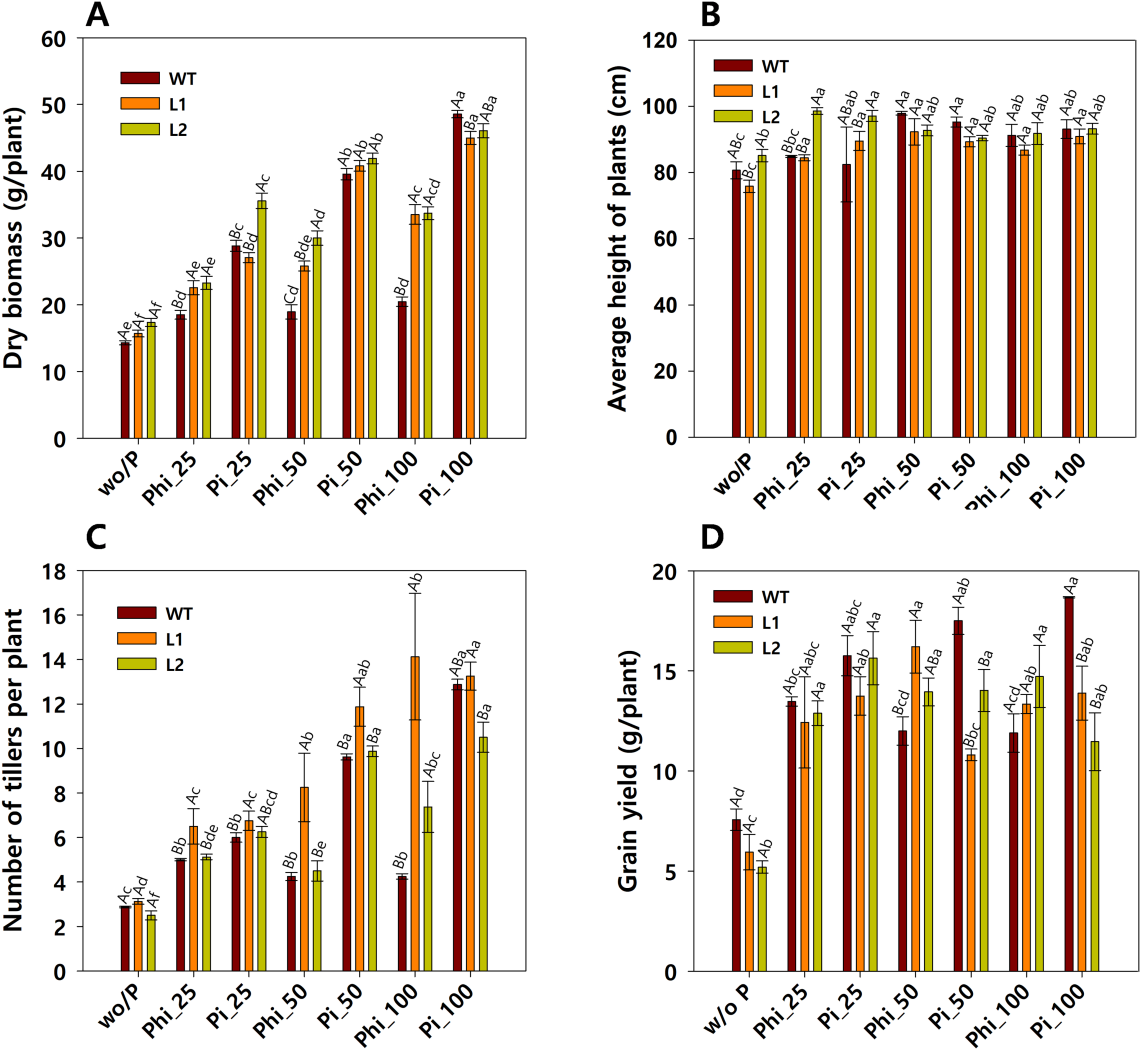
Biomass accumulation, plant height, tiller number, and grain yield of WT plants and ptxD lines grown in Ferralsol under different doses of phosphite (Phi) or phosphate (Pi). Fertilizer treatments corresponded to 0%, 25%, 50%, and 100% of the recommended phosphorus rate, equivalent to 0, 8.75, 17.5, and 35 kg ha^−1^ of elemental P, or 0, 4.36, 8.72, and 17.44 mg kg^−1^ of soil P (see Table S3 for detailed calculations of the Pi and Phi salt amounts applied per pot). Means followed by the same letters do not differ significantly. Capital letters compare genotypes within each treatment, whereas lowercase letters indicate differences among sources and doses within each genotype (Tukey's test, *p* ≤ 0.05).

At the end of the cultivation cycle, data were collected on biomass accumulation, plant height, tillering, and grain yield (Figure 3). At this stage, growth differences between ptxD lines cultivated with Phi and Pi were still apparent, although to a lesser extent. When grown with Pi, transgenic ptxD lines accumulated biomass comparable to that of WT plants at 25% and 50% of the recommended dose, and slightly lower at 100% (Figure 3A). These results indicate that the presence of the transgene does not compromise the ability of ptxD plants to utilize Pi for vegetative growth.

As expected, ptxD lines grown with Phi accumulated more biomass than WT plants, demonstrating the advantage conferred by *ptxD* expression in the presence of this compound. However, direct comparison of ptxD performance under Phi versus Pi supply revealed higher growth with Pi, suggesting that Pi uptake and/or utilization is more efficient than that of Phi. WT plants also showed increased biomass under Phi treatments compared to the phosphorus‐free control (Figure 3A), suggesting limited Phi utilization in soil, potentially mediated by microorganisms capable of converting Phi into Pi.

Regarding plant height (Figure 3B), differences were less pronounced than those observed for biomass accumulation. In contrast, tillering (Figure 3C) was more strongly influenced by the phosphorus source. Tiller number increased in both WT and ptxD lines with rising Pi doses. Under Phi cultivation, however, tiller number increased only in ptxD lines, reinforcing that only these transgenic plants can effectively utilize Phi as a phosphorus source. For grain yield (Figure 3D), WT plants grown with Phi produced more grains than those without phosphorus supply, suggesting that, even in the absence of the ptxD transgene, Phi might have been partially converted into Pi by soil microorganisms, potentially allowing plant uptake, although this remains speculative. Additionally, the positive yield response indicates that Phi did not exert any detectable toxic effects on these plants.

### 3.2 Performance of ptxD Plants Under Different Phi and Pi Concentrations in a Hydroponic System

The lower performance of ptxD lines grown in Ferralsol with Phi compared to Pi suggested a difference in uptake efficiency between these two phosphorus forms. To test this hypothesis, a hydroponic experiment was conducted with varying Pi and Phi concentrations to precisely evaluate plant response and nutrient uptake efficiency for each phosphorus source.

**FIGURE 4:**
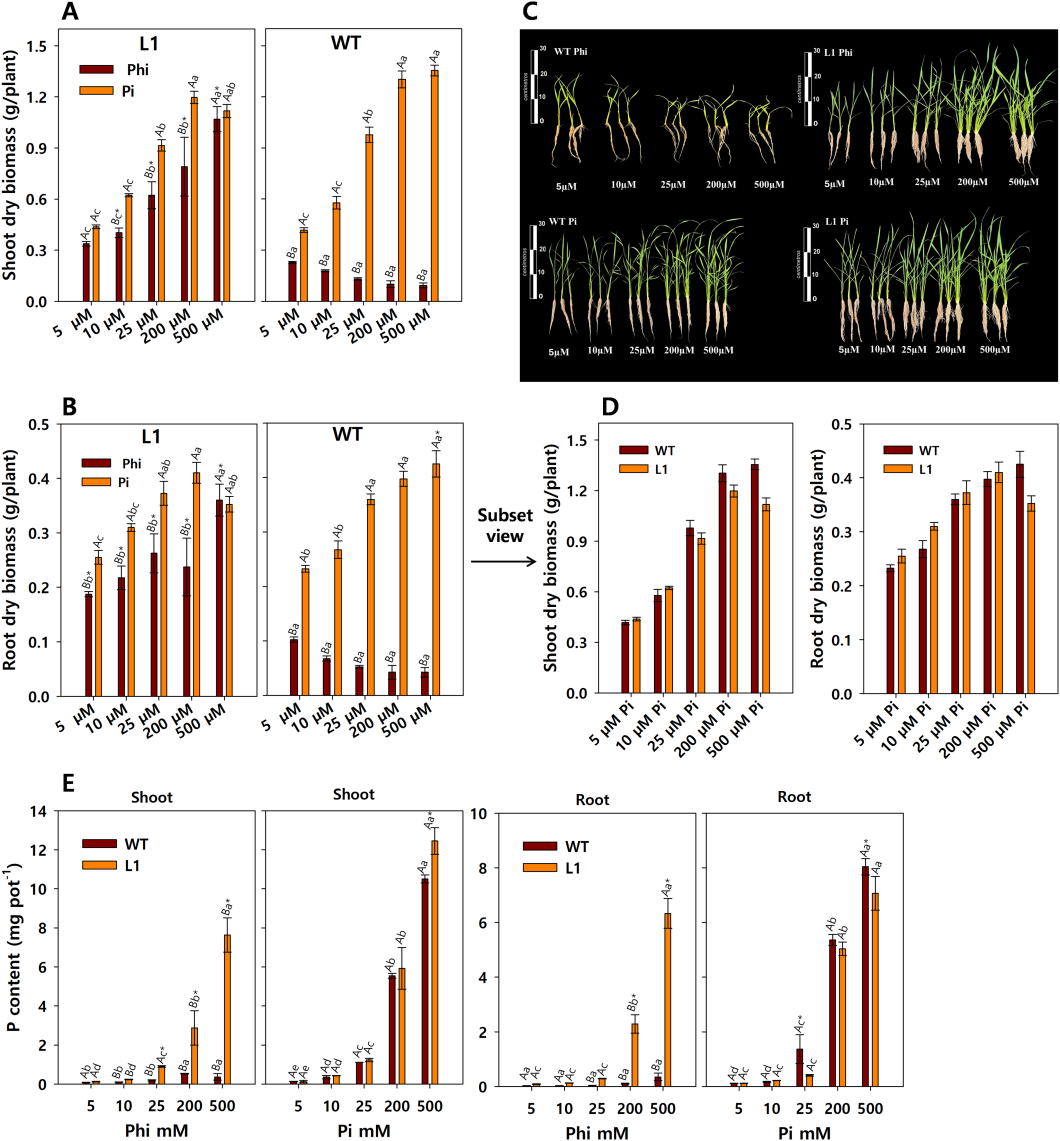
Performance of WT plants and the ptxD transgenic line (L1) grown in hydroponic culture with varying concentrations of phosphite (Phi) or phosphate (Pi). Plants were analyzed 28 days after germination. (A) Shoot dry biomass. (B) Root dry biomass. (C) Representative phenotype of WT and L1 plants grown under increasing Pi and Phi concentrations in hydroponic culture. (D) Subset view of the shoot and root biomass data highlighting the comparison between WT and L1 plants. (E) Total phosphorus content of WT and L1 plants grown under increasing Pi and Phi concentrations. Identical letters indicate no significant differences. In panels A and B, uppercase letters refer to phosphorus sources, lowercase letters refer to phosphorus concentrations, and * denotes the line that was statistically superior according to Tukey's test at the 5% significance level. In panel E, uppercase letters compare genotypes within each treatment, whereas lowercase letters compare Pi or Phi concentrations within each genotype, according to Tukey's test at the 5% significance level.

Data from the hydroponic assay indicate that, at the lowest phosphorus concentrations, shoot and root biomass accumulation in ptxD plants was lower under Phi compared to Pi supply. This difference suggests a limitation in Phi uptake or utilization under low‐availability conditions. However, this disparity disappeared at the highest concentration tested (500 μM), where biomass accumulation in ptxD plants was equivalent between the two phosphorus sources (Figure 4A,B). These results demonstrate that although transgenic plants are capable of absorbing and utilizing Phi, the process is less efficient than Pi uptake, particularly at low phosphorus concentrations.

Additionally, the hydroponic assay revealed that at lower Pi concentrations (10–25 μM), WT plants and ptxD lines exhibited comparable shoot and root biomass accumulation. At higher Pi concentrations (500 μM), however, WT plants accumulated more biomass than ptxD lines (Figure 4D).

Phosphorus accumulation was quantified in shoot and root tissues of WT and ptxD (L1) plants grown under increasing concentrations of Pi or Phi in the hydroponic system (Figure 4E). Under Pi supply, both genotypes exhibited a dose‐dependent increase in tissue P content, with comparable accumulation patterns in shoots and roots, indicating that the transgene does not alter Pi uptake capacity per se. In contrast, under Phi supply, WT plants maintained very low P levels across all concentrations, consistent with their inability to metabolize Phi. Conversely, ptxD plants showed a clear dose‐dependent increase in P accumulation in both shoots and roots, particularly at 200 and 500 mM Phi. The magnitude of P accumulation under Phi in ptxD plants paralleled their improved biomass production observed under the same conditions.

### 3.3 Expression of Phosphorus‐Deficiency Responsive Genes in WT and ptxD Plants Grown Hydroponically With Pi and Phi

**FIGURE 5:**
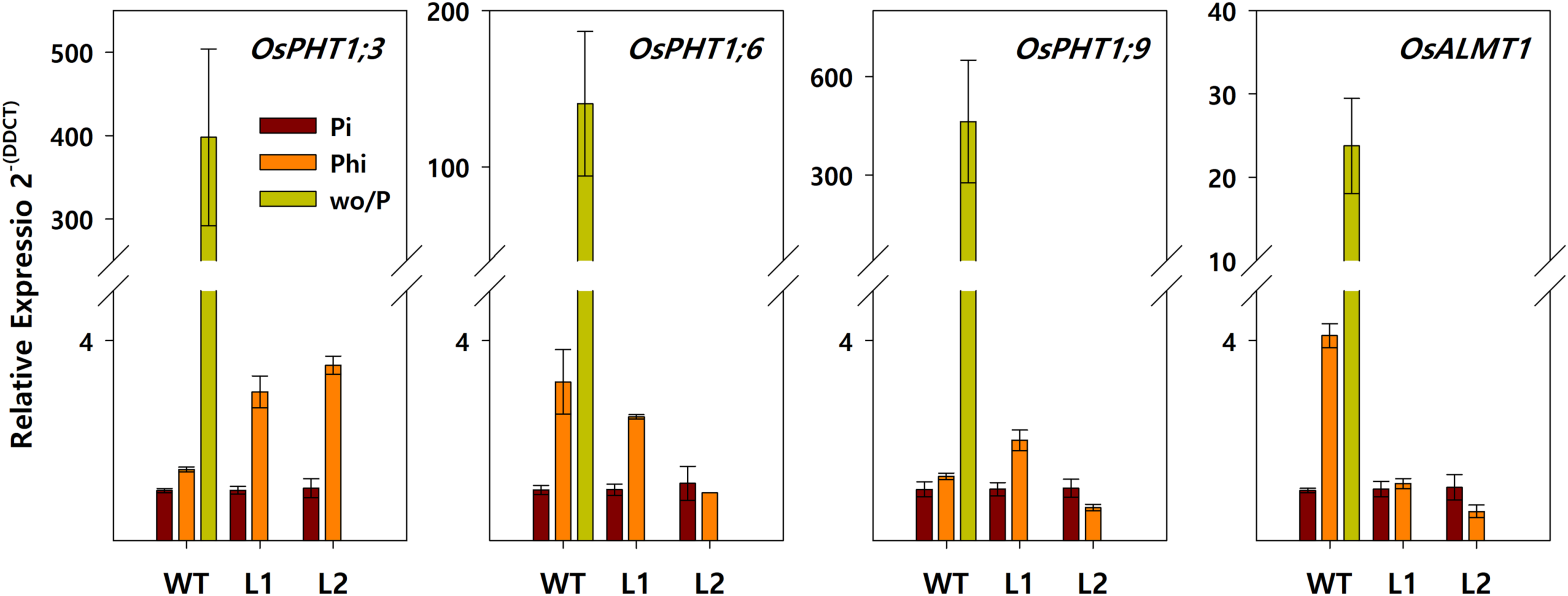
Relative expression of phosphorus‐deficiency responsive genes. Plants were grown until 14 days after germination in half‐strength Hoagland solution supplemented with 0.2 mM Phi or Pi. Additionally, WT plants were grown in phosphorus‐free solution as a nutrient‐deficiency control.

In this experiment, the expression of high‐affinity phosphate transporters and the malate transporter OsALMT1, all responsive to phosphorus deficiency, was evaluated (Seo et al. 2008; Balzergue et al. 2017; Liu et al. 2023). Although WT plants are incapable of metabolizing Phi, its presence strongly suppressed the typical transcriptional response to phosphorus deficiency (Figure 5). This observation suggests that Phi functions as a phosphate (Pi) mimic within the plant phosphorus signaling network, interfering with deficiency perception and the activation of genes involved in P homeostasis.

Nevertheless, the expression of OsPHT1;6 and OsALMT1 in WT plants grown with Phi was slightly elevated compared to phosphorus‐free conditions, albeit at much lower levels than under true P deficiency. In ptxD plants, Phi supply induced higher expression levels than Pi, particularly for OsPHT1;3, and in the L1 line, also for OsPHT1;6 and OsPHT1;9 (Figure 5).

### 3.4 Fluorometric Assay for Phosphite Quantification

**FIGURE 6:**
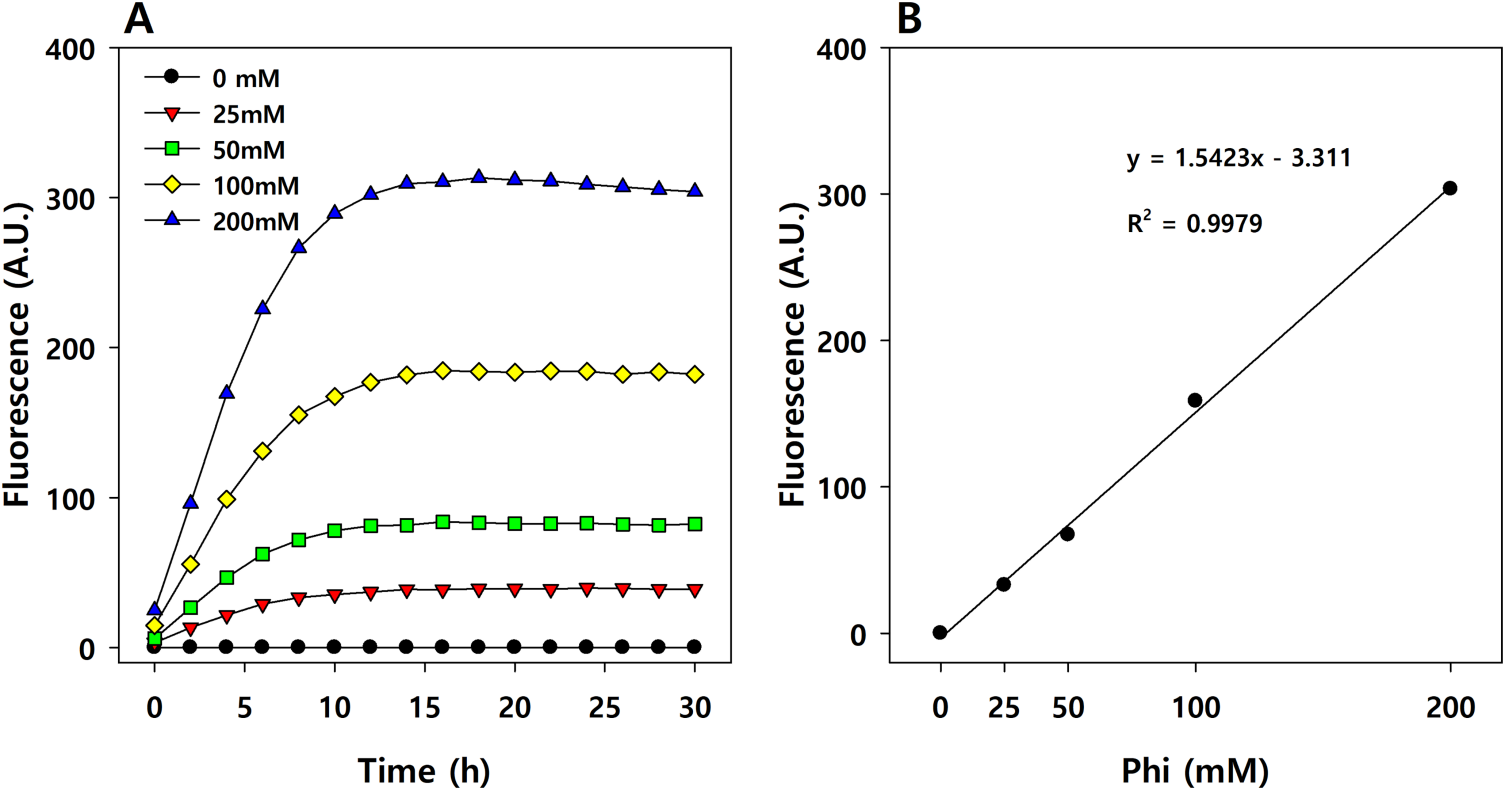
Assay conditions using 17X‐PTDH. (A) Real‐time detection of resorufin production in the enzymatic assay with increasing phosphite concentrations. (B) Standard curve for phosphite quantification. The reaction was incubated at 37°C for 30 min.

The kinetics of the enzymatic reaction over time for different Phi concentrations (0, 25, 50, 100, and 200 μM) are shown in Figure 6A. Fluorescence intensity, corresponding to resorufin formation, increased rapidly after the reaction was initiated for all Phi concentrations tested. Using 1 μg of 17X‐PTDH, the reaction reached saturation, with the fluorescent signal remaining stable after approximately 15 min across all Phi concentrations.

For Phi quantification, a standard curve was constructed with Phi concentrations ranging from 0 to 200 μM. Fluorescence values for each concentration were calculated as the difference between the signal at reaction saturation and that of the control solution (without Phi) measured at the same time point. Analysis was based on the endpoint of the reaction curve. A strong linear correlation between fluorescence intensity and Phi concentration was observed, with a coefficient of determination (*R* ^2^) of 0.9979, ensuring high accuracy in the quantification of Phi in nutrient solution samples.

### 3.5 Phosphorus Depletion in WT and ptxD Lines

**FIGURE 7:**
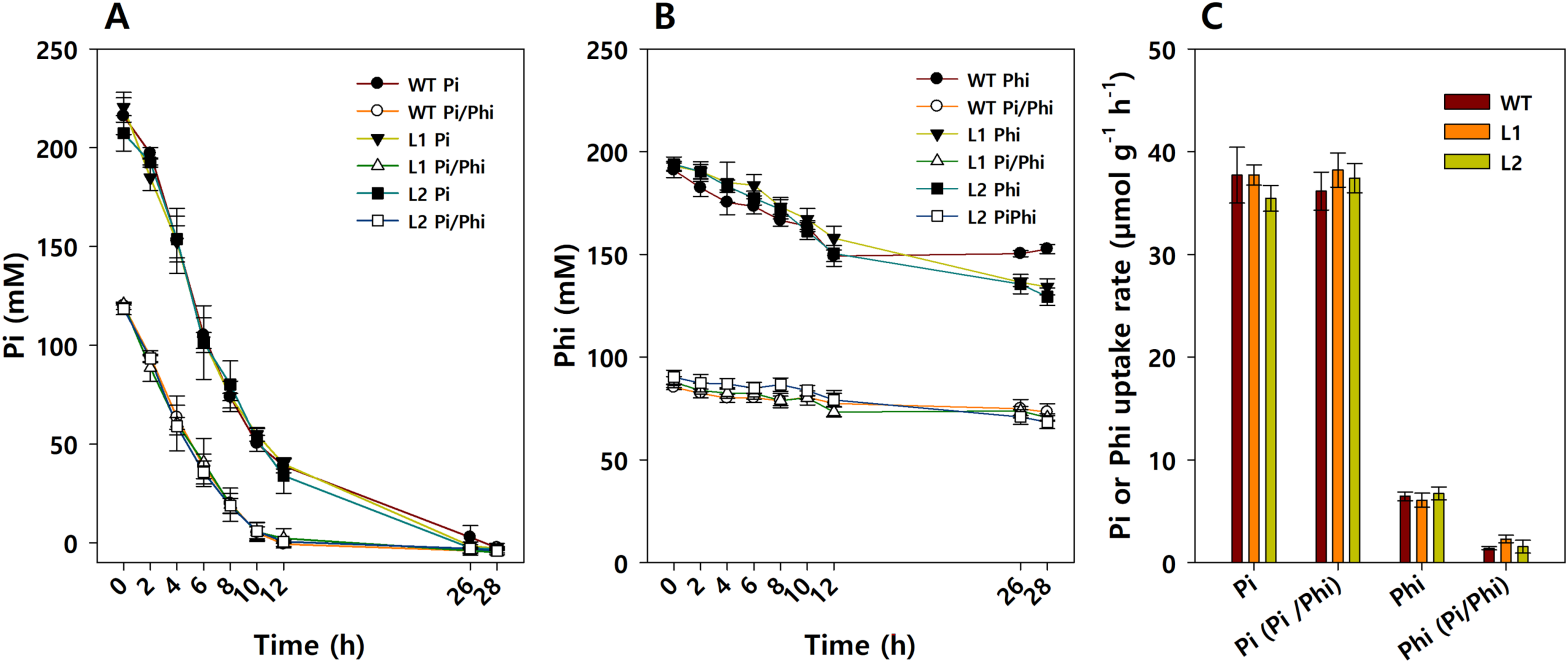
Depletion kinetics of Pi and Phi in nutrient solution over 28 h for WT, L1, and L2 plants. (A) Pi depletion in plants supplied exclusively with Pi at 0.2 mM and in combination with Pi/Phi at 0.1 mM each. (B) Phi depletion in plants supplied exclusively with Phi at 0.2 mM and in combination with Pi/Phi at 0.1 mM each. (C) Pi or Phi uptake rate (µmol g^−1^ h^−1^) in WT, L1, and L2 plants supplied exclusively with Pi or Phi (0.2 mM) and under combined Pi/Phi conditions (0.1 mM each). Values represent means ± SE.

In this assay, plants were subjected to 6 days of phosphorus deficiency, a period sufficient to induce the expression of high‐affinity transporters and enhance nutrient uptake upon resupply. Analysis of depletion kinetics revealed significantly higher absorption rates for Pi compared to Phi in both WT and ptxD lines (Figure 7A,B), indicating that although both forms of phosphorus can be absorbed, transporters exhibit greater efficiency for Pi uptake.

In solutions containing only Pi or Phi at 0.2 mM, Pi was absorbed rapidly, whereas Phi was depleted more slowly over time. Notably, a clear distinction between WT and ptxD lines in Phi uptake was observed only after 26 h (Figure 7B). When Pi and Phi were supplied simultaneously (0.1 mM each), Pi was rapidly absorbed, whereas Phi remained largely unchanged, suggesting a preferential uptake of Pi in the presence of both sources, or alternatively, that Phi absorption is inherently more limited at low concentrations.

The Pi uptake rate was similar under both conditions, 0.2 mM Pi alone or a Pi/Phi mixture containing 0.1 mM of each, with no differences observed between transgenic lines and WT plants (Figure 7C). In contrast, Phi uptake was strongly influenced by the presence of Pi. Plants supplied with Phi alone exhibited higher uptake rates than those grown in the Pi/Phi mixture, indicating that Pi competitively inhibits Phi uptake.

## 4 Discussion

The introduction of the ptxD gene into rice enabled the utilization of Phi as an alternative phosphorus source, corroborating previous findings in other species (López‐Arredondo and Herrera‐Estrella 2012; Manna et al. 2016; Xu et al. 2024). In the present study, transgenic lines exhibited a clear growth advantage over WT plants under Phi supply. Tissue P quantification further provided direct biochemical evidence of functional Phi utilization, as ptxD plants displayed significant, dose‐dependent P accumulation under Phi, whereas WT plants failed to accumulate P or sustain growth. Because these experiments were conducted under hydroponic conditions, where eventual microbial conversion of Phi to Pi is negligible, the increased tissue P content and associated enhanced growth observed in transgenic plants relative to WT can be attributed to transgene‐mediated oxidation of Phi into metabolically available Pi. Together, these results substantiate the central premise that ptxD expression enables effective Phi utilization as a phosphorus source.

However, depletion kinetics revealed an important limitation in the system. Even after a period of phosphorus deprivation sufficient to induce high‐affinity transporter expression, the Phi uptake rate was substantially lower than that of Pi in both WT and transgenic plants. This result aligns with classical studies indicating direct competition between Pi and Phi for the same transporters (Danova‐Alt et al. 2008; Achary et al. 2017). Furthermore, when both forms were present, Pi was rapidly absorbed while Phi remained nearly constant in solution, suggesting a higher affinity of the transporters for Pi or, alternatively, competitive restriction of Phi entry at low concentrations (Figure 7C). These findings reinforce the idea that the main limitation of the system is not only the conversion of Phi to Pi catalyzed by ptxD but also the uptake of Phi by plant cells.

In the Ferralsol experiment, the inferior performance of ptxD plants under Phi may be associated with both limited uptake kinetics and edaphic factors. The soil used, prior to lime correction, exhibited high acidity and elevated exchangeable aluminum, properties known to intensify phosphorus adsorption (Vinha et al. 2021; Lambers 2022). Although Phi is generally described as less reactive with Fe and Al oxides (Manna et al. 2016), its intrinsically lower uptake efficiency may have reduced its effective availability to plants.

Biomass increases were observed in WT plants cultivated with Phi, which could suggest the possibility of gradual Phi oxidation occurring in the soil over the 5‐month cultivation period under long‐day conditions. Previous studies have reported the existence of soil microorganisms capable of oxidizing Phi to Pi (Ewens et al. 2021). However, no direct measurements of microbial activity or Phi‐to‐Pi conversion were performed in the present study; therefore, microbial involvement cannot be confirmed. The markedly reduced early growth of WT plants under Phi supply indicates that, if microbial oxidation occurred, it was likely slow and insufficient to meet plant P demand during initial developmental stages. Thus, any contribution of soil‐mediated Phi conversion should be considered hypothetical within the scope of this work.

Gene expression results demonstrated that Phi acts as a molecular mimic of Pi, repressing the typical phosphorus‐deficiency response in WT plants. This effect has been previously described in Arabidopsis (Varadarajan et al. 2002; Jost et al. 2015) and explains the reduced expression of high‐affinity transporters under Phi, even when plants are metabolically unable to utilize it. It is important to emphasize that phosphorus starvation typically triggers a very strong transcriptional activation of phosphate transporter genes, often reaching increases of a 100‐fold compared with P‐sufficient conditions (Figure 5). Consequently, the relatively small differences in transcript abundance observed among WT and transgenic lines under Phi supply should be interpreted with caution. Such variations are minor compared with the magnitude of induction associated with genuine P deficiency and may not necessarily reflect biologically meaningful differences in phosphorus acquisition or signaling, but rather subtle differences in the internal P status of the plants.

The ALMT1 gene, originally cloned from an Al‐resistant wheat ( *Triticum aestivum* ) line, encodes an anion channel (TaALMT1) responsible for Al‐activated malate efflux from root apices (Sasaki et al. 2004). In soybean, the ortholog GmALMT1 regulates malate exudation and contributes to improved adaptation to acidic soils (Liang et al. 2013). In previous analyses, we observed that *OsALMT1* is also responsive to phosphorus deficiency, with WT plants showing an approximately 20‐fold induction under P starvation and a ~4‐fold increase under Phi supply. In contrast, the transgenic lines did not show detectable changes in *OsALMT1* expression under Phi. This pattern suggests that, although WT plants did not strongly induce high‐affinity phosphate transporters under Phi, they may have activated alternative mechanisms to mobilize phosphorus in the rhizosphere.

Under hydroponic conditions, the performance of ptxD plants under Phi matched that under Pi only at the highest concentration tested (500 μM), indicating that the limitation is primarily manifested at low doses. This pattern has important agronomic implications, as the ptxD/phi system is promising for selective weed management, conferring a competitive advantage to transgenic plants (López‐Arredondo and Herrera‐Estrella 2012; Xu et al. 2024). However, Phi alone does not constitute a viable substitute for Pi in low‐fertility tropical soils.

From a practical perspective, these results indicate that Phi can be used complementarily with conventional phosphate fertilization, partially reducing Pi use and expanding integrated nutrient and weed management options. Nevertheless, its application as a complete phosphate substitute is limited by absorption barriers and adverse edaphic conditions, particularly in highly weathered soils where P availability is naturally constrained.

In conclusion, this study highlights that the limited root uptake of Phi, relative to phosphate, constitutes a major bottleneck to its use as a primary phosphorus source in ptxD transgenic plants. Overcoming this challenge may require the development of transporters with higher Phi affinity, as well as integrating the ptxD/phi strategy with broader management practices that combine conventional phosphate fertilization with complementary Phi application, either to enhance phosphorus nutrition or to exploit its selective effects on invasive plants and Phi‐sensitive pathogens.

## Author Contributions

This study is a collaborative effort of all authors. C.C.L.d.O., M.d.F.L., and L.A.S. conceptualized and designed the experiments. C.C.L.d.O., A.L.d.S.P.N., J.N.d.O.S., T.P.d.S., M.E.P.d.M., C.A.d.S.J., and I.G.N. performed the experiments and conducted data analysis. C.C.L.d.O. and L.A.S. wrote the original draft and finalized the manuscript. N.C.d.S., G.L.L., and M.d.F.L. contributed to specific experiments and measurements. L.A.S., A.C.G., and M.d.F.L. provided resources and project support. L.A.S. supervised the research and performed critical review and editing of the manuscript. All authors read, contributed to, and approved the final manuscript.

## Funding

This work was supported by the Rio de Janeiro Research Foundation (FAPERJ) and the National Council for Scientific and Technological Development (CNPq). The article processing charge (APC) was funded by the Coordination for the Improvement of Higher Education Personnel (CAPES).

## Supporting information


**Figure S1:** Schematic representation of the native and codon‐optimized ptxD gene from *Pseudomonas stutzeri* for expression in *Oryza sativa* ssp. *japonica*. Codon optimization was performed using the GenSmart Codon Optimization tool (GenScript). Nucleotide substitutions in the optimized sequence relative to the native gene are highlighted in red.
**Figure S2:** Schematic representation of the genetic construct used for rice transformation. (A) Expression vector IRS154_ptxD, in which the *ptxD* gene is driven by the maize ubiquitin 1 promoter (ZmUbi), ensuring strong and constitutive expression in rice. (B) Detailed representation of the genetic elements integrated into the rice genome.
**Figure S3:** Confirmation of genetic transformation using primers targeting different segments of the construct. (A) Amplification of a fragment of the hygromycin phosphotransferase (hpt) gene. (B) Amplification of the region spanning the maize ubiquitin promoter (ZmUbi) and the nopaline synthase terminator (t‐NOS). (C) Amplification of a fragment of the ptxD gene. M, 1 kb DNA Ladder (Avatti); L1–L2, transgenic rice lines; +, positive PCR control.
**Figure S4:** Schematic representation of the pot experiment using Ferralsol soil. Seeds of homozygous (T2) rice lines were germinated in water and, after 7 days, transplanted into pots containing 8 kg of soil. Nitrogen fertilization was applied at 90 kg ha^−1^ and potassium at 60 kg ha^−1^, following the guidelines of the Soil Fertility Manual (Freire 2013). Phosphorus treatments were established on an elemental P basis at 0, 8.75, 17.5, and 35 kg ha^−1^ of P (equivalent to 0%, 25%, 50%, and 100% of the recommended rate). These rates corresponded to 0, 4.36, 8.72, and 17.44 mg kg^−1^ of soil P (see Table S3 for detailed calculations of the Pi and Phi salt amounts applied per pot).
**Table S1:** Primers used to confirm transgenic rice plants. HPT was employed to amplify the hygromycin phosphotransferase (hpt) gene, which confers resistance to hygromycin; ptxD3 was used to amplify a fragment of the ptxD gene; and ZmUbi‐F/Tnot‐R was designed to amplify the region spanning the promoter and terminator of the genetic construct.
**Table S2:** List of primers used for the expression analysis of the ptxD gene and genes associated with phosphorus transport and phosphorus deficiency response in rice plants.
**Table S3:** Stoichiometric calculation of salt mass required to supply equivalent elemental P rates in Pi and Phi treatments.

## Data Availability

All data supporting the findings of this study are available within the manuscript and its Supporting Information files.

## References

[ppl70969-bib-0001] Achary, V. M. M. , B. Ram , M. Manna , et al. 2017. “Phosphite: A Novel P Fertilizer for Weed Management and Pathogen Control.” Plant Biotechnology Journal 15: 1493–1508. 10.1111/pbi.12803. 28776914 PMC5698055

[ppl70969-bib-0002] Bailey, C. A. , and B. L. Greene . 2023. “A Fluorometric Assay for High‐Throughput Phosphite Quantitation in Biological and Environmental Matrices.” Analyst 148: 3650–3658. 10.1039/D3AN00575E. 37424451

[ppl70969-bib-0003] Balzergue, C. , T. Dartevelle , C. Godon , et al. 2017. “Low Phosphate Activates STOP1‐ALMT1 to Rapidly Inhibit Root Cell Elongation.” Nature Communications 8: 15300. 10.1038/ncomms15300. PMC5440667 28504266

[ppl70969-bib-0004] Costas, A. M. G. , A. K. White , and W. W. Metcalf . 2001. “Purification and Characterization of a Novel Phosphorus‐Oxidizing Enzyme From *Pseudomonas stutzeri* WM88.” Journal of Biological Chemistry 276: 17429–17436. 11278981 10.1074/jbc.M011764200

[ppl70969-bib-0005] Cutolo, E. , M. Tosoni , S. Barera , L. Herrera‐Estrella , L. Dall'Osto , and R. Bassi . 2020. “A Phosphite Dehydrogenase Variant With Promiscuous Access to Nicotinamide Cofactor Pools Sustains Fast Phosphite‐Dependent Growth of Transplastomic *Chlamydomonas reinhardtii* .” Plants 9: 473. 10.3390/plants9040473. 32276527 PMC7238262

[ppl70969-bib-0006] da Silva, O. F. , and A. E. Wander . 2025. “Importância Econômica e Social.” In Agência Embrapa de Informação Tecnológica: arroz, edited by EMBRAPA . EMBRAPA. https://www.embrapa.br/agencia‐de‐informacao‐tecnologica/cultivos/arroz/pre‐producao/socioeconomia/importancia‐economica‐e‐social.

[ppl70969-bib-0007] Danova‐Alt, R. , C. O. R. Dijkema , P. de Waard , and M. Köck . 2008. “Transport and Compartmentation of Phosphite in Higher Plant Cells—Kinetic and 31P Nuclear Magnetic Resonance Studies.” Plant, Cell & Environment 31: 1510–1521. 10.1111/j.1365-3040.2008.01861.x. 18657056

[ppl70969-bib-0008] Ewens, S. D. , A. F. Gomberg , T. P. Barnum , et al. 2021. “The Diversity and Evolution of Microbial Dissimilatory Phosphite Oxidation.” Proceedings of the National Academy of Sciences of The United States of America 118: e2020024118. 10.1073/pnas.2020024118. 33688048 PMC7980464

[ppl70969-bib-0009] FAO . 2022. Crop Prospects and Food Situation. Vol. 4. Food and Agriculture Organization of the United Nations.

[ppl70969-bib-0010] Freire, L. R. 2013. “Manual de Calagem e Adubação do Estado do Rio de Janeiro.”

[ppl70969-bib-0011] Gao, J. , J. Liu , B. Li , and Z. Li . 2001. “Isolation and Purification of Functional Total RNA From Blue‐Grained Wheat Endosperm Tissues Containing High Levels of Starches and Flavonoids.” Plant Molecular Biology Reporter 19: 185–186.

[ppl70969-bib-0012] Han, Z. , J. Shi , J. Pang , L. Yan , P. M. Finnegan , and H. Lambers . 2021. “Foliar Nutrient Allocation Patterns in *Banksia attenuata* and *Banksia sessilis* Differing in Growth Rate and Adaptation to Low‐Phosphorus Habitats.” Annals of Botany 128: 419–430. 10.1093/aob/mcab013. 33534909 PMC8414927

[ppl70969-bib-0013] Holsters, M. , D. De Waele , A. Depicker , E. Messens , M. Van Montagu , and J. Schell . 1978. “Transfection and Transformation of *Agrobacterium tumefaciens* .” Molecular & General Genetics 163: 181–187. 10.1007/BF00267408. 355847

[ppl70969-bib-0014] Howe, G. W. , and W. A. Van Der Donk . 2019. “Temperature‐Independent Kinetic Isotope Effects as Evidence for a Marcus‐Like Model of Hydride Tunneling in Phosphite Dehydrogenase.” Biochemistry 58: 4260–4268. 10.1021/acs.biochem.9b00732. 31535852 PMC6852621

[ppl70969-bib-0015] IUSS Working Group WRB . 2022. World Reference Base for Soil Resources. International Soil Classification System for Naming Soils and Creating Legends for Soil Maps. 4th ed. International Union of Soil Sciences (IUSS).

[ppl70969-bib-0016] Jost, R. , M. Pharmawati , H. R. Lapis‐Gaza , et al. 2015. “Differentiating Phosphate‐Dependent and Phosphate‐Independent Systemic Phosphate‐Starvation Response Networks in *Arabidopsis thaliana* Through the Application of Phosphite.” Journal of Experimental Botany 66: 2501–2514. 10.1093/jxb/erv025. 25697796 PMC4986860

[ppl70969-bib-0017] Juo, A. S. , and K. Franzluebbers . 2003. Tropical Soils: Properties and Management for Sustainable Agriculture. Oxford University Press.

[ppl70969-bib-0018] Lambers, H. 2022. “Phosphorus Acquisition and Utilization in Plants.” Annual Review of Plant Biology 73: 17–42. 10.1146/annurev-arplant-102720-125738. 34910587

[ppl70969-bib-0019] Liang, C. , M. A. Piñeros , J. Tian , et al. 2013. “Low pH, Aluminum, and Phosphorus Coordinately Regulate Malate Exudation Through GmALMT1 to Improve Soybean Adaptation to Acid Soils.” Plant Physiology 161, no. 3: 1347–1361. 10.1104/pp.112.208934. 23341359 PMC3585601

[ppl70969-bib-0020] Liu, T. , S. Deng , C. Zhang , et al. 2023. “Brassinosteroid Signaling Regulates Phosphate Starvation‐Induced Malate Secretion in Plants.” Journal of Integrative Plant Biology 65: 1099–1112. 10.1111/jipb.13443. 36579777

[ppl70969-bib-0040] Liu, T. , L. Yuan , S. Deng , et al. 2021. “Improved the Activity of Phosphite Dehydrogenase and Its Application in Plant Biotechnology.” Frontiers in Bioengineering and Biotechnology 9: 764188. 10.3389/fbioe.2021.764188. 34900961 PMC8655118

[ppl70969-bib-0021] Livak, K. J. , and T. D. Schmittgen . 2001. “Analysis of Relative Gene Expression Data Using Real‐Time Quantitative PCR and the 2−ΔΔCT Method.” Methods 25: 402–408. 10.1006/meth.2001.1262. 11846609

[ppl70969-bib-0022] López‐Arredondo, D. L. , and L. Herrera‐Estrella . 2012. “Engineering Phosphorus Metabolism in Plants to Produce a Dual Fertilization and Weed Control System.” Nature Biotechnology 30: 889–893. 10.1038/nbt.2346. 22922674

[ppl70969-bib-0023] Luz, J. H. S. , and L. E. M. Brito . 2022. “A eficiência do Uso de Fósforo Pode Ser Melhorada com o uso de Substâncias Húmicas? Uma Revisão.” Agricultural and Environmental Sciences 8: 13. 10.36725/agries.v8i2.7928.

[ppl70969-bib-0024] Manna, M. , V. M. M. Achary , T. Islam , P. K. Agrawal , and M. K. Reddy . 2016. “The Development of a Phosphite‐Mediated Fertilization and Weed Control System for Rice.” Scientific Reports 6: 24941. 10.1038/srep24941. 27109389 PMC4842969

[ppl70969-bib-0025] Navea, I. P. , S. Yang , P. Tolangi , R. M. Sumabat , W. Zhang , and J. H. Chin . 2024. “Enhancement of Rice Traits for the Maintenance of the Phosphorus Balance Between Rice Plants and the Soil.” Current Plant Biology 37: 100332. 10.1016/j.cpb.2024.100332.

[ppl70969-bib-0027] Pantano, G. , G. M. Grosseli , A. A. Mozeto , and P. S. Fadini . 2016. “Sustentabilidade no uso do Fósforo: Uma Questão de Segurança Hídrica e Alimentar.” Química Nova 39: 732–740. 10.5935/0100-4042.20160086.

[ppl70969-bib-0028] R Core Team . 2020. R: A Language and Environment for Statistical Computing. R Foundation for Statistical Computing.

[ppl70969-bib-0029] Roberts, T. L. , and A. E. Johnston . 2015. “Phosphorus Use Efficiency and Management in Agriculture.” Resources, Conservation and Recycling 105: 275–281. 10.1016/j.resconrec.2015.09.013.

[ppl70969-bib-0030] Sasaki, T. , Y. Yamamoto , B. Ezaki , et al. 2004. “A Wheat Gene Encoding an Aluminum‐Activated Malate Transporter.” Plant Journal 37: 645–653. 10.1111/j.1365-313X.2003.01991.x. 14871306

[ppl70969-bib-0031] Seo, H. M. , Y. Jung , S. Song , et al. 2008. “Increased Expression of *OsPT1*, a High‐Affinity Phosphate Transporter, Enhances Phosphate Acquisition in Rice.” Biotechnology Letters 30: 1833–1838. 10.1007/s10529-008-9757-7. 18563580

[ppl70969-bib-0032] Tedesco, M. J. , C. Gianello , C. A. Bissani , H. Bohnen , and S. J. Volkweiss . 1995. Análise de Solo, Plantas e Outros Materiais. Vol. 5, 174. UFRGS, Departamento de Solos, Boletim Técnico.

[ppl70969-bib-0033] Teixeira, P. C. , G. K. Donagemma , A. Fontana , and W. G. Teixeira . 2017. Manual de Métodos de Análise de Solo. Embrapa.

[ppl70969-bib-0034] Toki, S. , N. Hara , K. Ono , et al. 2006. “Early Infection of Scutellum Tissue With *Agrobacterium* Allows High‐Speed Transformation of Rice.” Plant Journal 47: 969–976. 10.1111/j.1365-313X.2006.02836.x. 16961734

[ppl70969-bib-0035] USDA . 2023. “World Agricultural Production.” Circular Series. WAP 8–23. August 2023. https://apps.fas.usda.gov/psdonline/circulars/production.pdf.

[ppl70969-bib-0036] Varadarajan, D. K. , A. S. Karthikeyan , P. D. Matilda , and K. G. Raghothama . 2002. “Phosphite, an Analog of Phosphate, Suppresses the Coordinated Expression of Genes Under Phosphate Starvation.” Plant Physiology 129: 1232–1240. 10.1104/pp.010835. 12114577 PMC166517

[ppl70969-bib-0037] Vinha, A. P. C. , B. H. Carrara , E. F. S. Souza , J. A. F. dos Santos , and S. A. C. M. Arantes . 2021. “Adsorção de Fósforo em Solos de Regiões Tropicais.” Nativa 9: 30–35. 10.31413/nativa.v9i1.10973.

[ppl70969-bib-0038] White, A. K. , and W. W. Metcalf . 2007. “Microbial Metabolism of Reduced Phosphorus Compounds.” Annual Review of Microbiology 61: 379–400. 10.1146/annurev.micro.61.080706.093357. 18035609

[ppl70969-bib-0039] Xu, D. , T. Xiong , W. Lu , J. Zhao , Z. Zhang , and G. Xiao . 2024. “The *ptxD* Gene Confers Rapeseed the Ability to Utilize Phosphite and a Competitive Advantage Against Weeds.” Agronomy 14: 727. 10.3390/agronomy14040727.

[ppl70969-bib-0041] Yang, X. , and W. A. van der Donk . 2015. “Post‐Translational Introduction of D‐Alanine into Ribosomally Synthesized Peptides by the Dehydroalanine Reductase NpnJ.” Journal of the American Chemical Society 137, no. 39: 12426–12429. 10.1021/jacs.5b05207. 26361061 PMC4599312

