# Supplementary material for: Can Phosphite Substitute for Phosphate in ptxD Rice? Evidence From Uptake and Agronomic Performance
Source: Physiol Plant. 2026 Jun 19;178(3):e70969. doi: 10.1111/ppl.70969 (PMC13282367; doi:10.1111/ppl.70969)
Supplement: Supplementary file 1 — Figure S1: Schematic representation of the native and codon‐optimized ptxD gene from Pseudomonas stutzeri for expression in Oryza sativa ssp. japonica. Codon optimization was performed using the GenSmart Codon Optimization tool (GenScript). Nucleotide substitutions in the optimized sequence relative to the native gene are highlighted in red. Figure S2: Schematic representation of the genetic construct used for rice transformation. (A) Expression vector IRS154_ptxD, in which the ptxD gene is driven by the maize ubiquitin 1 promoter (ZmUbi), ensuring strong and constitutive expression in rice. (B) Detailed representation of the genetic elements integrated into the rice genome. Figure S3: Confirmation of genetic transformation using primers targeting different segments of the construct. (A) Amplification of a fragment of the hygromycin phosphotransferase (hpt) gene. (B) Amplification of the region spanning the maize ubiquitin promoter (ZmUbi) and the nopaline synthase terminator (t‐NOS). (C) Amplification of a fragment of the ptxD gene. M, 1 kb DNA Ladder (Avatti); L1–L2, transgenic rice lines; +, positive PCR control. Figure S4: Schematic representation of the pot experiment using Ferralsol soil. Seeds of homozygous (T2) rice lines were germinated in water and, after 7 days, transplanted into pots containing 8 kg of soil. Nitrogen fertilization was applied at 90 kg ha−1 and potassium at 60 kg ha−1, following the guidelines of the Soil Fertility Manual (Freire 2013). Phosphorus treatments were established on an elemental P basis at 0, 8.75, 17.5, and 35 kg ha−1 of P (equivalent to 0%, 25%, 50%, and 100% of the recommended rate). These rates corresponded to 0, 4.36, 8.72, and 17.44 mg kg−1 of soil P (see Table S3 for detailed calculations of the Pi and Phi salt amounts applied per pot). Table S1: Primers used to confirm transgenic rice plants. HPT was employed to amplify the hygromycin phosphotransferase (hpt) gene, which confers resistance to hygromycin; ptxD3 was used [file PPL-178-e70969-s001.docx]

**Supplementary Material**

**Can Phosphite Substitute for Phosphate in PtxD Rice? Evidence from Uptake and Agronomic Performance**

Clenya Carla Leandro de Oliveira¹; André Luís da Silva Parente Nogueira¹; José Nivaldo de Oliveira Sátiro¹; Tiago Paula da Silva¹; Maria Eduarda Pimentel de Melo¹; Castro Alves da Silva Junior¹; Izabela Gouveia Nascimento¹; Natália Corrêa da Silva^2,3^; Gabriel Lima Leal³; Marcelo de Freitas Lima³; Andrés Calderín Garcia¹; Leandro Azevedo Santos¹.

¹ Institute of Agronomy, Department of Soil Science, Federal Rural University of Rio de Janeiro, Seropédica, Brazil

² Institute of Biomedical Sciences, Federal University of Rio de Janeiro, Rio de Janeiro, Brazil

³ Institute of Chemistry, Department of Biochemistry, Federal Rural University of Rio de Janeiro, Seropédica, Brazil

**Figure S1.** Schematic representation of the native and codon-optimized ptxD gene from Pseudomonas stutzeri for expression in Oryza sativa ssp. japonica. Codon optimization was performed using the GenSmart™ Codon Optimization tool (GenScript). Nucleotide substitutions in the optimized sequence relative to the native gene are highlighted in red.


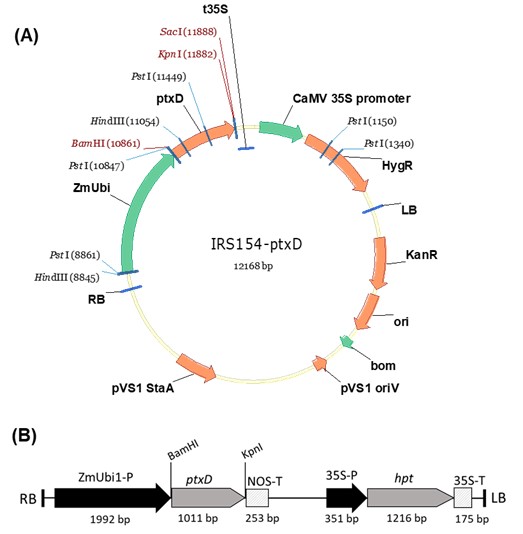


**Figure S2.** Schematic representation of the genetic construct used for rice transformation. (A) Expression vector IRS154_ptxD, in which the *ptxD* gene is driven by the maize ubiquitin 1 promoter (ZmUbi), ensuring strong and constitutive expression in rice. (B) Detailed representation of the genetic elements integrated into the rice genome.


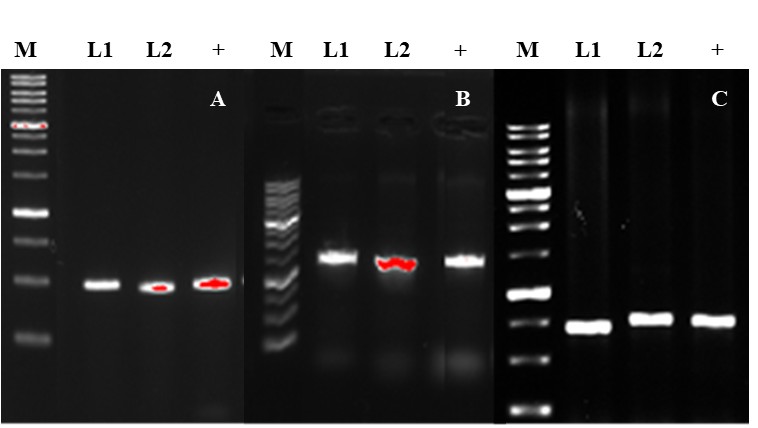


**Figure S3.** Confirmation of genetic transformation using primers targeting different segments of the construct. (A) Amplification of a fragment of the hygromycin phosphotransferase (hpt) gene. (B) Amplification of the region spanning the maize ubiquitin promoter (ZmUbi) and the nopaline synthase terminator (t-NOS). (C) Amplification of a fragment of the ptxD gene. M, 1 kb DNA Ladder (Avatti); L1–L2, transgenic rice lines; +, positive PCR control.


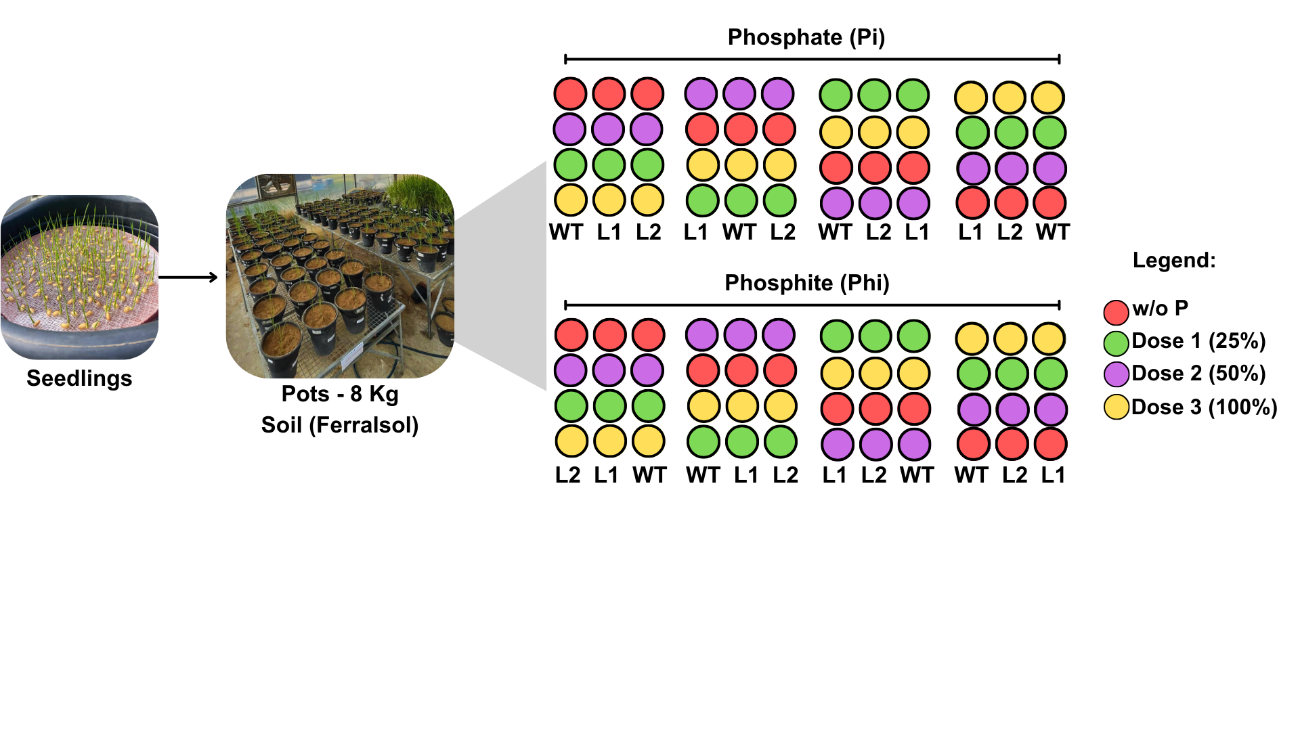


**Figure S4.** Schematic representation of the pot experiment using Ferralsol soil. Seeds of homozygous (T2) rice lines were germinated in water and, after 7 days, transplanted into pots containing 8 kg of soil. Nitrogen fertilization was applied at 90 kg ha⁻¹ and potassium at 60 kg ha⁻¹, following the guidelines of the Soil Fertility Manual (Freire, 2013). Phosphorus treatments were established on an elemental P basis at 0, 8.75, 17.5, and 35 kg ha⁻¹ of P (equivalent to 0%, 25%, 50%, and 100% of the recommended rate). These rates corresponded to 0, 4.36, 8.72, and 17.44 mg kg⁻¹ of soil P (see Supplementary Table S3 for detailed calculations of the Pi and Phi salt amounts applied per pot).

**Table S1.** Primers used to confirm transgenic rice plants. HPT was employed to amplify the hygromycin phosphotransferase (hpt) gene, which confers resistance to hygromycin; ptxD3 was used to amplify a fragment of the ptxD gene; and ZmUbi-F/Tnot-R was designed to amplify the region spanning the promoter and terminator of the genetic construct.

| **Target** | **Primer sequence (5’-3’)** |
| --- | --- |
| **HPTF** | CCAACCACGGCCTCCAGAAGAAGATGT |
| **HPTR** | TTGGGGAGTTTAGCGAGAGCCTGACCT |
| **Ptxd3F** | GATCCTTCGCCGTTGTAGAG |
| **Ptxd3R** | TTGACGAGGTGCTGAGTGTC |
| **ZmUbi-F** | TTTAGCCCTGCCTTCATACG |
| **t-NOS-R** | AAGACCGGCAACAGGATTC |

**Table S2.** List of primers used for the expression analysis of the ptxD gene and genes associated with phosphorus transport and phosphorus deficiency response in rice plants.

| **Gene** | **Real time PCR primers (5'–3')** | **Locus MSU 7** |
| --- | --- | --- |
| *OsALMT1* | For: AGCTGCCAAAACACTCAGGA | LOC_Os04g34010 |
|  | Rev: CGTGCCAGGTTATCGACAGA |  |
| *OsPT3* | For: GAGGAGCAAGACGAACGAGG | LOC_Os10g30770 |
|  | Rev: TCCAGCGTCGTCAGTACCTT |  |
| *OsPT6* | For: TGTTCTCCGGCGAGTTTGTC | LOC_Os08g45000 |
|  | Rev: CTGGAACAGGTTCTGGCTGT |  |
| *OsPT9* | For: GCGCTTCCACGAACTAAATGT | LOC_Os06g21920 |
|  | Rev: AGGATAGGCGACATGCTGAG |  |
| *PtxD* | For: GCATCATCGGACTTCATCCT | GenBank: AF061070.1 |
|  | Rev: CTAACCAACGCCAGAAGCTC |  |
| *OsActin1* | For: CTTCATAGGAATGGAAGCTGCGGGTA | LOC_Os03g50885 |
|  | Rev: CGACCACCTTGATCTTCATGCTGCTA |  |
| *OsUBQ5* | For: ACCACTTCGACCGCCACTACT | LOC_Os01g22490 |
|  | Rev: ACGCCTAAGCCTGCTGGTT |  |

**Table S3**. Stoichiometric calculation of salt mass required to supply equivalent elemental P rates in Pi and Phi treatments

| **Target P dose (kg ha⁻¹)** | **Equivalent P (mg kg⁻¹ soil)** | **P fraction in salt** | **Salt required (mg kg⁻¹ soil)** | **P source (salt)** |
| --- | --- | --- | --- | --- |
| 0 | 0.00 | 0.218 | 0 | Na₂HPO₄ (Pi) |
| 8.75 | 4.36 | 0.218 | 20 | Na₂HPO₄ (Pi) |
| 17.5 | 8.72 | 0.218 | 40 | Na₂HPO₄ (Pi) |
| 35 | 17.44 | 0.218 | 80 | Na₂HPO₄ (Pi) |
| 0 | 0.00 | 0.1434 | 0.00 | Na₂HPO₃·5H₂O (Phi) |
| 8.75 | 4.36 | 0.1434 | 30.41 | Na₂HPO₃·5H₂O (Phi) |
| 17.5 | 8.72 | 0.1434 | 60.82 | Na₂HPO₃·5H₂O (Phi) |
| 35 | 17.44 | 0.1434 | 121.64 | Na₂HPO₃·5H₂O (Phi) |

Pi was supplied as Na₂HPO₄ (anhydrous), molar mass = 141.956 g mol⁻¹. Fraction of P = 30.974 / 141.956 = 0.2182 (21.8%).

Phi was supplied as Na₂HPO₃·5H₂O, molar mass = 216.032 g mol⁻¹. Fraction of P = 30.974 / 216.032 = 0.1434 (14.34%).

Salt mass was calculated as:

$$Salt (mg kg⁻¹)=\frac{Target P (mg \mathrm{kg}^{-1}soil)}{P fraction in \mathrm{salt}}$$

Conversion from kg ha⁻¹ to mg kg⁻¹ soil followed the soil mass equivalence adopted in the pot experiment.
